# Cross-Reactivity and Cross-Intolerance Among Nonsteroidal Anti-Inflammatory Drugs (NSAIDs): Clinical Patterns, COX-1-Mediated Mechanisms, and Implications for COX-2 Inhibitors and Paracetamol

**DOI:** 10.3390/ijms27093727

**Published:** 2026-04-22

**Authors:** Wiktoria Andryszkiewicz, Martyna Lippik, Małgorzata Makieła, Bartosz Modrzyk, Krzysztof Gomułka

**Affiliations:** 1 Research Group of Allergology and Internal Medicine, Wroclaw Medical University, 50-556 Wroclaw, Poland; wiktoria.andryszkiewicz@student.umw.edu.pl (W.A.); martyna.lippik@student.umw.edu.pl (M.L.); malgorzata.makiela@student.umw.edu.pl (M.M.); bartosz.modrzyk@student.umw.edu.pl (B.M.); 2 Clinical Department of Allergology and Internal Medicine, Wroclaw Medical University, 50-556 Wroclaw, Poland

**Keywords:** nonsteroidal anti-inflammatory drugs, cross-reactivity, hypersensitivity, cyclooxygenase inhibitors, paracetamol, bioactive compounds

## Abstract

Cross-reactivity among nonsteroidal anti-inflammatory drugs (NSAIDs) creates a significant clinical difficulty, especially in patients with NSAID hypersensitivity. These reactions are based on cyclooxygenase-1 (COX-1) inhibition and non-immunoglobulin E (IgE)-mediated reactions. COX-1 inhibition leads to dysregulation of arachidonic acid metabolism, with decreased prostaglandin synthesis and increased leukotriene production. Clinically, cross-intolerant reactions manifest in different phenotypes, including NSAID-exacerbated respiratory disease (NERD), NSAID-induced urticaria/angioedema (NIUA), and NSAID-exacerbated cutaneous disease (NECD). In contrast, true allergic reactions—such as single-NSAID-induced urticaria/angioedema and anaphylaxis (SNIUAA) and single-NSAID-induced delayed hypersensitivity reactions (SNIDHR)—are immunologically mediated and drug-specific. These phenotypes differ in underlying conditions, clinical manifestations, and patterns of NSAID tolerance. Paracetamol is generally considered a safer alternative due to its weak COX-1 inhibition; however, reactions may still occur, particularly at higher doses. Selective COX-2 inhibitors are usually better tolerated, however their safety should be confirmed, preferably through controlled drug provocation testing due to sporadic reactions in cross-intolerant patients. Understanding the distinction between pharmacologically mediated cross-intolerance and true allergic reactions is essential for accurate diagnosis, risk stratification, and therapeutic decision-making. This review summarizes current evidence on the mechanisms underlying NSAID hypersensitivity, analyzes the tolerability of paracetamol and alternative analgesics, and discusses practical management strategies to reduce the risk of adverse reactions.

## 1. Introduction

Nonsteroidal anti-inflammatory drugs (NSAIDs) are known as one of the most commonly used medications in the world, and they present anti-inflammatory, antipyretic, and analgesic effects [1,2]. Their over-the-counter availability may cause the common use of NSAIDs. As stated in a telephone survey, almost half of the examined people with chronic pain were taking non-prescription analgesics—55% were taking NSAIDs, 43% paracetamol, and 13% weak opioids [3].

NSAIDs inhibit prostaglandin biosynthesis through cyclooxygenase enzymes (COX), which transform arachidonic acid into various forms of prostaglandins. Prostaglandins are mediators of pain, inflammation, and temperature regulation. COX may occur as isoenzymes COX-1 and COX-2. The COX-1 enzyme is expressed in most cells and plays a role in protecting the gastric mucosa, maintaining renal function, and maintaining homeostasis. On the contrary, inflammation and cancer can induce COX-2. Additionally, NSAIDs’ cytotoxic effects on cells, induced by mitochondrial oxidative stress (MOS), have recently been studied. Paracetamol has similar analgesic and antipyretic activity, but lacks anti-inflammatory activity [4,5].

The prevalence of hypersensitivity to NSAIDs has been higher only in antibiotics, but in some centers, the reactions to NSAIDS were the most common. The overall prevalence of hypersensitivity to NSAIDs falls within the ambit of 0.6 to 5.7% of the general population, but depending on the studied population, results may vary [6]. Hypersensitivity reactions to NSAIDs should be distinguished from classical immune-mediated drug allergy. In this context, most reactions are non-immunologic and related to COX-1 inhibition, whereas a smaller proportion represents true allergic, drug-specific responses [7]. The manifestation of hypersensitivity is an adverse drug reaction (ADR), defined as a harmful or unintended reaction to a drug that occurs at doses used for prevention, diagnosis, or treatment. ADR is classified into two categories: type A, which is predictable, dose-dependent, and related to the drug’s known pharmacological action; and type B, which is unpredictable and unrelated to the drug’s known pharmacological action. A drug allergy is defined as a type B ADR. The risk factors of a drug allergy can be put into two categories: patient-related or drug-related. Patient-related factors include viral infections (Human Immunodeficiency Virus, Epstein–Barr virus, herpes viruses), genetic polymorphisms, young age, and female sex. To drug-related factors, we count the route of administration and dose. Ergo, topical administration is associated with the highest risk, followed by intravenous or intramuscular, and oral administration is associated with the lowest risk. Additionally, frequent use is associated with a higher risk than a single dose [8].

**Figure 1:**
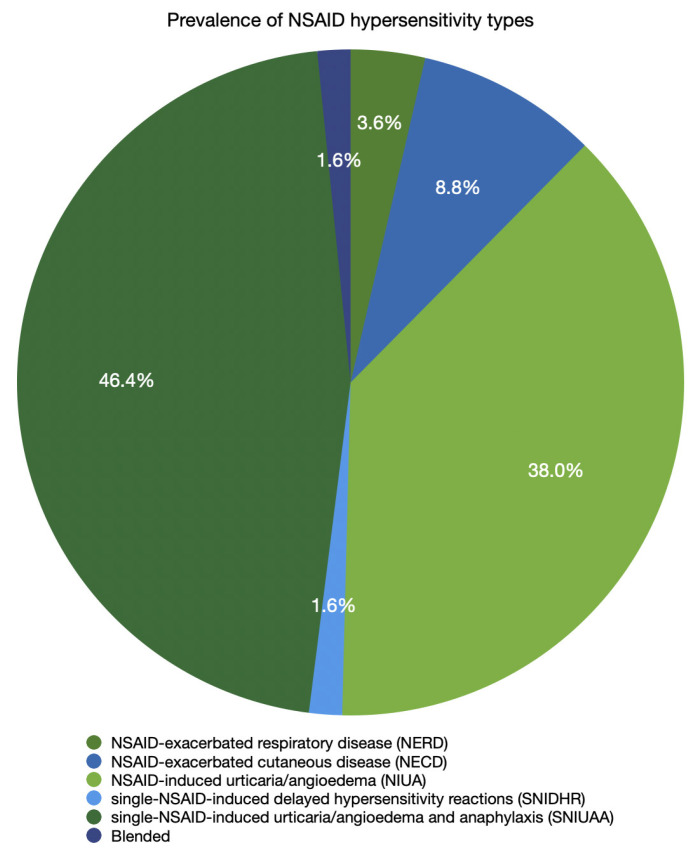
The prevalence of NSAID hypersensitivity types (based on [10,11]).

An important aspect of hypersensitivity reactions is cross-reactivity, which occurs when a drug not previously administered elicits a reaction due to preexisting sensitization to a structurally related compound or to common pharmacological characteristics. While many antibiotics’ cross-reactivities are explained by the presence of a common antigenic determinant in the cross-reacting drugs, cross-reactivity among NSAIDs is primarily explained by COX-1-mediated cross-intolerance rather than structural or immunologic cross-reactivity [9]. NSAID hypersensitivity may manifest with diverse clinical symptoms, and the classification system for it was developed by the European Academy of Allergy & Clinical Immunology (EAACI). It recognizes 5 phenotypes based on the timing of symptom onset, the presence of underlying disease, and the involvement of organ systems. Classification includes NSAID-exacerbated respiratory disease (NERD), NSAID-exacerbated cutaneous disease (NECD), NSAID-induced urticaria/angioedema (NIUA), single-NSAID-induced urticaria/angioedema and anaphylaxis (SNIUAA), and single-NSAID-induced delayed hypersensitivity reactions (SNIDHR) [10]. The prevalence of these types in a total of 308 patients with confirmed NSAID hypersensitivity is shown in Figure 1 [10,11]. The prevalence of NSAID hypersensitivity is higher in patients with bronchial asthma and nasal polyps. And so, hypersensitivity to aspirin, one of the NSAIDs, affects from 0.5% to 1.9% of the general population and from 4.3% to 11% of adult asthmatic patients [12]. Additionally, hypersensitivity to aspirin, one of the NSAIDs, has been associated with a variety of genetic polymorphisms causing leukotriene overproduction, eosinophil infiltration, and histamine-related genes [13,14].

## 2. Mechanisms of Action and Pathophysiology of Cross-Reactive Responses

The first NSAID, acetylsalicylic acid (ASA), and later other NSAIDs have been prescribed for years due to their analgesic, anti-inflammatory, and platelet-inhibitory effects. Although these medications offer many benefits, some predisposed patients report NSAID intolerance and side effects, which are mostly associated with COX-1 enzyme inhibition [14]. Asthmatic patients are particularly predisposed and are at high risk of developing ASA-exacerbated respiratory disease (AERD, also known as NERD) as well as other ASA-induced respiratory reactions, such as rhinitis, nasal polyposis, and anosmia [15]. It has been reported that 10–20% people with asthma developed AERD [16]. Moreover, NSAIDs, which inhibit COX-1, such as ibuprofen, indomethacin, diclofenac, and naproxen, also induce asthmatic attacks and tend to react with ASA [17].

Hypersensitivity between ASA and other NSAIDs is mainly based on two phenotypes: the previously mentioned AERD and aspirin-intolerant urticaria (AIU), which belongs to the NIUA category. AIU can be further divided into two subgroups: aspirin-intolerant acute urticaria (AIAU) and aspirin-intolerant chronic urticaria (AICU) [13]. Both AIAU and AICU are considered subtypes of NIUA, reflecting non-immunologic cross-intolerance reactions primarily mediated by COX-1 inhibition, rather than IgE-mediated allergy. The overproduction of cysteinyl leukotrienes (CysLTs) and increased expression of their receptors in the respiratory mucosa, together with decreased synthesis of lipoxins and prostaglandin E2 (PGE2), are crucial in the pathogenesis of AERD. Increased activity of major biosynthetic enzymes—5-lipoxygenase and leukotriene C4 synthase—potentially of genetic origin, has been suggested to underlie the excessive production of CysLTs in patients with aspirin-sensitive asthma. Mast cells and eosinophils are responsible for CysLT production and are more abundant in biopsy samples from patients with aspirin-sensitive asthma. CysLTs and leukotriene B4 (LTB4) act via separate high-affinity G protein-coupled receptors, namely CysLT1 and CysLT2, and additionally via the LTB4 receptor. The majority of the proinflammatory effects of CysLTs are mediated through activation of the CysLT1 receptor. Moreover, macrophages, T cells, eosinophils, mast cells, and neutrophils also express leukotriene receptors [18,19].

Although the mechanism of AICU is still not fully explained, immunologic pathways, including IgE-mediated pathways, may contribute in a minority of patients. However, AICU is generally considered part of the cross-reactive, COX-1-mediated hypersensitivity spectrum rather than a classical IgE-mediated drug allergy. Genetic polymorphisms in high-affinity IgE receptors, especially FcεR1β E237G and FcεR1γ 237G, are associated with a higher rate of atopy in patients with chronic urticaria, predisposing them to AICU. These receptors are expressed on mast cells and basophils and can trigger IgE-mediated allergic responses, leading to the release of histamine and proinflammatory cytokines [20]. Moreover, in most AICU patients, mast cell activation and urticarial reactions are primarily driven by COX-1 inhibition and dysregulation of arachidonic acid metabolism, rather than by adaptive immune mechanisms. Patients with chronic urticaria also show elevated mast cell tryptase and urinary LTE4 levels compared with healthy individuals. Both aspirin and COX-1-inhibiting NSAIDs (e.g., naproxen) can provoke cutaneous urticarial reactions accompanied by increased urinary LTE4 excretion, further supporting the central role of COX-1 inhibition in AICU [21]. Table 1 presents key mediators and factors influencing NSAID hypersensitivity reactions.

Non-immunologic, cross-reactive hypersensitivity reactions primarily depend on the degree of COX-1 inhibition, whereas immunologic (selective) reactions are related to drug-specific chemical structures and immune recognition mechanisms, primarily the presence or absence of a sulfonamide group. Drugs with sulfonamide groups are selective COX-2 inhibitors (coxibs), including celecoxib, etoricoxib, and parecoxib. Inhibition of COX-1 shifts arachidonic acid metabolism toward the lipoxygenase pathway, leading to decreased prostaglandin synthesis and increased CysLT production. This mechanism is pharmacological rather than immunologic and does not involve drug-specific IgE antibodies. Consequently, these reactions are referred to as non-allergic hypersensitivity or cross-intolerance, rather than true drug allergy. The N1 heterocyclic ring, together with an arylamine substituent at N4 in sulfonamide drugs, is associated with hypersensitivity reactions promoting IgE-mediated mast cell activation. Although most sulfonamide NSAIDs lack this chemical feature, the effects of other chemical structures within the sulfonamide group are responsible for hypersensitivity reactions. This process may lead to tissue injury or stimulate cellular or humoral immunity, involving T-cell-mediated responses, in response to haptenation or antigens [22,23]. Moreover, among all chemical groups of NSAIDs, heteroaryl acetic acids, including diclofenac, tolmetin, and ketorolac, present a higher risk of NSAID-induced anaphylactic reaction. Arylpropionic acids, including ibuprofen, naproxen, ketoprofen, and fenoprofen, may also cause anaphylactic reactions; however, the risk is lower [24].

Additionally, poorly selective NSAIDs showed more hypersensitivity reactions in comparison with coxibs. Coxibs are also safer to use in terms of hospitalization due to angioedema and are better tolerated by people who present urticaria [25,26,27]. Nevertheless, a recent report suggests inconsistent outcomes, as many patients experiencing NSAID-induced urticaria/angioedema could also be sensitive to etoricoxib, particularly in individuals who presented cross-intolerance to acetaminophen [28].

## 3. Clinical Presentation and Reactivity Patterns

As mentioned before, hypersensitivity reactions to NSAIDs are classified into allergic (immunologically mediated) and non-allergic (non-immunologic) reactions based on clinical features, underlying pathophysiology, and patterns of cross-reactivity. EAACI distinguishes five phenotypes according to the timing of symptom onset, the presence of underlying diseases, and the organs involved. Non-immunologic phenotypes include NERD, NECD, and NIUA, whereas immunologic reactions comprise single-NSAID-induced SNIUAA and SNIDHR [10,29].

The most prevalent NSAID hypersensitivity phenotypes are non-immunologic and result from pharmacological inhibition of COX-1. Reduced PGE2 synthesis shifts arachidonic acid metabolism toward the 5-lipoxygenase pathway, increasing cysteinyl leukotriene production and promoting bronchoconstriction, vascular permeability, and inflammation. This mechanism explains the typical respiratory manifestations in NERD and cutaneous symptoms in NIUA [30,31]. In contrast, allergic NSAID hypersensitivity reactions are immune-mediated, drug-specific, and independent of COX-1 inhibition. They may be immediate, mediated by drug-specific IgE antibodies and presenting as urticaria, angioedema, or anaphylaxis, or delayed, mediated by T lymphocytes and manifesting as various cutaneous eruptions. These reactions are usually limited to a single NSAID or a chemically related group and do not show cross-reactivity with structurally unrelated agents [32,33].

### 3.1. NERD

NERD is a non-allergic, cross-intolerance phenotype of NSAID hypersensitivity characterized by respiratory symptoms occurring after exposure to NSAIDs that inhibit COX-1. Clinical manifestations typically develop within 30 min to 3 h and include bronchospasm, wheezing, dyspnea, chest tightness, as well as nasal congestion, rhinorrhea, and conjunctival symptoms. This phenotype predominantly affects patients with preexisting chronic airway diseases, most commonly asthma and chronic rhinosinusitis with nasal polyps (CRSwNP). Many patients report a history of progressive asthma severity and recurrent sinonasal symptoms preceding NSAID intolerance. Reactions occur with multiple chemically unrelated NSAIDs, reflecting a cross-intolerance pattern [34,35]. From a mechanistic perspective, reactions in NERD are driven by pharmacological intolerance related to COX-1 inhibition and subsequent dysregulation of eicosanoid balance, rather than immunologic sensitization. As a result, patients usually exhibit cross-reactivity to multiple structurally unrelated NSAIDs with strong COX-1 inhibitory activity [11,35].

### 3.2. NECD

NECD is a non-allergic, cross-reactive phenotype observed in patients with preexisting chronic spontaneous urticaria or recurrent urticaria. In this group, NSAID exposure leads to worsening or reactivation of cutaneous symptoms, including urticaria and/or angioedema. Symptoms usually appear within 1–6 h following drug ingestion and are limited primarily to the skin. Importantly, NECD does not cause new-onset urticaria, but rather exacerbates an existing skin condition. Patients often report variable disease activity, with NSAIDs acting as a reproducible trigger during active phases of chronic spontaneous urticaria [6,36].

The underlying mechanism of NECD is pharmacological and resembles that observed in other cross-intolerance NSAID hypersensitivity phenotypes. occurring within the first hour of drug ingestion, although both immediate reactions (within 15 min) and delayed reactions (onset of symptoms after several hours). By this mechanism, patients typically tolerate weak COX-1 inhibitors, such as paracetamol, and preferential drugs, such as meloxicam and nimesulide, or selective COX-2 inhibitors [36].

### 3.3. NIUA

NIUA is one of the most frequently reported NSAID hypersensitivity phenotypes in both adults and children and is defined by acute urticaria and/or angioedema occurring after NSAID intake in patients who do not have chronic spontaneous urticaria or other baseline cutaneous disease. This phenotype is considered a non-allergic, cross-intolerance reaction mediated by COX-1 inhibition [6,35]. Clinically, NIUA presents with wheals, itchiness, and localized or generalized angioedema, occurring within the first hour of drug ingestion, although both immediate reactions and delayed reactions may occur. Mild respiratory or systemic manifestations may occasionally accompany these symptoms, but do not reflect the underlying chronic cutaneous or respiratory disease seen in NECD or NERD, respectively. NIUA patients are typically sensitive to multiple chemically unrelated NSAIDs that inhibit COX-1. Weak COX-1 inhibitors may cause symptoms in some patients, especially if used in high doses such as 1000 mg of paracetamol. Selective COX-2 inhibitors are usually well tolerated [28,37].

### 3.4. SNIUAA

SNIUAA defines a selective immunologic hypersensitivity phenotype in which reactions occur only to a specific NSAID or chemically related group, while other NSAIDs are tolerated. The response is independent of COX-1 inhibition potency, suggesting a drug-specific immunological mechanism [6]. These reactions usually occur within the first hour following drug intake. However, this interval can be longer, and symptoms may range from skin reactions, such as urticaria or angioedema, to generalized erythema to anaphylaxis [38]. The pathogenic mechanism of SNIUAA is closely related to classical IgE-mediated allergic reactions to other drugs, such as antibiotics. The mechanism is independent of COX-1 inhibition and is most often associated with drug-specific IgE-mediated or other immune pathways, which explains the absence of cross-reactivity with structurally unrelated NSAIDs [10].

### 3.5. SNIDHR

SNIDHR encompasses delayed reactions occurring hours to several days after exposure to a specific NSAID. The clinical spectrum includes maculopapular exanthema, fixed drug eruptions, contact dermatitis, and, in rare cases, severe cutaneous adverse reactions (SCARs) such as Stevens–Johnson syndrome or toxic epidermal necrolysis. Blood dyscrasias such as anemia, agranulocytosis, and thrombocytopenia have rarely been observed. [39,40,41]. Unlike cross-reactive patterns, these reactions are mediated by immune pathways and selectively involve a single drug or related [39,42].

### 3.6. Paracetamol

Paracetamol is generally considered a safer analgesic alternative in patients with NSAID hypersensitivity; however, a subset of patients exhibits hypersensitivity reactions to paracetamol, particularly those with cross-intolerance NSAID intolerance phenotypes [6]. Drug provocation tests with paracetamol induced symptoms in 11 out of 49 patients (22.4%) diagnosed with NERD and in 3 of 32 patients (9.4%) with NIUA [43]. In another study, 6 out of 105 patients with NSAID hypersensitivity (5.7%) had cross-reaction to paracetamol [44]. Importantly, the outcome of paracetamol challenge appears to be strongly dose-dependent. Children aged 9–14 with confirmed NIUA tolerated low doses of paracetamol (approximately 15 mg/kg) well [45]. In contrast, in a pediatric cohort of seven children under 12 years of age who exhibited positive reactions to ASA and tolerated a cumulative paracetamol dose of 80 mg, two children developed hypersensitivity symptoms when the dose was increased to 240 mg [46]. The tolerance of paracetamol is primarily attributed to its weak inhibition of cyclooxygenase-1, which is insufficient to trigger clinical symptoms in many patients at standard doses. Nevertheless, at higher doses, paracetamol may inhibit COX-1 to a degree sufficient to provoke reactions in susceptible individuals. In contrast to cross-intolerance, patients with immunologically mediated, selective NSAID hypersensitivity usually tolerate paracetamol well, as these reactions are independent of cyclooxygenase-1 inhibition and lack cross-reactivity with structurally unrelated analgesics [47]. Table 2 compares NSAID hypersensitivity phenotypes.

Non-allergic NSAID hypersensitivity reactions are characterized by a predictable, dose-dependent response related to COX-1 inhibition and typically occur in patients with specific underlying conditions, such as asthma, chronic rhinosinusitis with nasal polyps, or chronic spontaneous urticaria. These reactions usually develop shortly after drug intake and recur upon exposure to multiple chemically unrelated NSAIDs with strong COX-1 inhibitory activity, reflecting a cross-intolerance pattern [48]. In contrast, allergic NSAID hypersensitivity reactions are immune-mediated, selective, and independent of COX-1 inhibition. They result from drug-specific immune sensitization, involving IgE antibodies in immediate reactions or T-cell-mediated mechanisms in delayed reactions. They usually occur after exposure to a single NSAID or a chemically related group. They may present as immediate IgE-mediated reactions, such as urticaria, angioedema, or anaphylaxis, or as delayed T-cell-mediated reactions manifesting as various cutaneous eruptions [49]. Accurate differentiation between non-allergic, COX-1-dependent intolerance and true allergic NSAID hypersensitivity is essential for clinical management. While cross-intolerance reactions necessitate avoidance of all strong COX-1 inhibitors, selective allergic reactions require targeted avoidance of the culprit drug and enable the safe use of alternative NSAIDs under appropriate supervision [33].

## 4. Management Strategies and Therapeutic Alternatives

**Figure 2:**
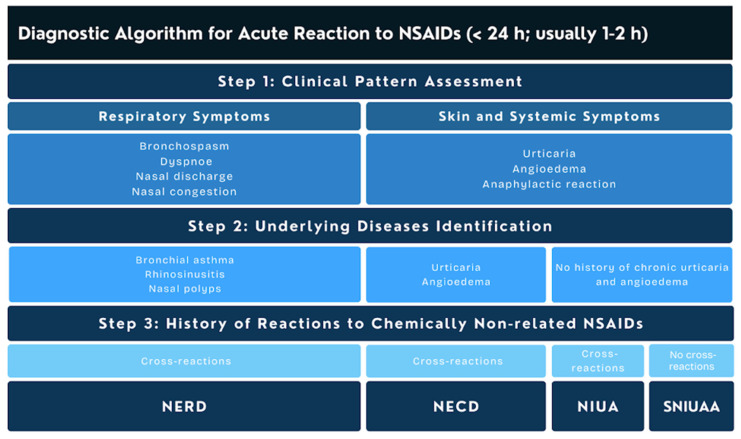
Diagnostic algorithm for acute reaction to nonsteroidal anti-inflammatory drugs (NSAIDs). NSAID–exacerbated respiratory disease (NERD), NSAID–exacerbated cutaneous disease (NECD), NSAID–induced urticaria/angioedema (NIUA), single-NSAID–induced urticaria/angioedema and anaphylaxis (SNIUAA).

Hypersensitivity to ASA and other NSAIDs represents a prevalent clinical challenge in contemporary clinical practice for drug hypersensitivity [50]. Definitive diagnosis is predicated on a stratified diagnostic approach and the implementation of validated algorithms. These protocols encompass a comprehensive clinical history, in vitro assays, and/or controlled provocation challenges utilizing either the culprit agent or a structurally unrelated alternative drug, contingent upon the specific reaction phenotype. The preliminary diagnosis is primarily established through a rigorous anamnesis of prior drug hypersensitivity events [12]. This clinical record must encapsulate critical parameters, including a detailed characterization of the symptom spectrum, the chronological onset (latency period), the specific brand and pharmacological class of the culprit drug, the dosage, the route of administration, and a list of concomitant medications. Of particular diagnostic significance is the frequency of episodes induced by chemically distinct NSAIDs. Patients presenting with ≥3 episodes elicited by different NSAIDs, including potent COX-1 inhibitors, are classified as exhibiting cross-intolerance related to COX-1 inhibition. In such cases, the specific phenotype is determined by the underlying comorbidities and the nature of the clinical manifestations experienced post-ingestion [39]. Conversely, if the patient has experienced <3 episodes involving <3 distinct NSAIDs, the oral provocation test (OPT) with aspirin remains the diagnostic gold standard. This procedure demonstrates high diagnostic accuracy, with a documented negative predictive value (NPV) of 97.8% and a positive predictive value (PPV) approaching 100% [50]. Figure 2 presents the diagnostic algorithm for acute NSAID reaction.

Despite its high diagnostic yield, the OPT protocol possesses significant clinical drawbacks. It is highly labor-intensive, typically requiring a minimum of two days for completion, and demands stringent monitoring due to the risk of inducing severe iatrogenic bronchospasm. Consequently, OPT is contraindicated in patients with a history of severe bronchospasm or unstable bronchial asthma. In these cohorts, alternative provocation modalities, such as nasal or bronchial provocation tests, should be prioritized. Skin testing with the culprit agent is considered of limited utility and is indicated only when the clinical history suggests SNIUAA.

Over the last decade, in vitro assays measuring NSAID-specific peripheral blood leukocyte activation have been proposed as alternative diagnostic procedures, particularly for patients at high risk of severe hypersensitivity reactions. While numerous studies have evaluated their efficacy, the reported sensitivity and specificity remain variable and inconsistent. Furthermore, these assays are unavailable for the majority of NSAIDs and frequently yield false-negative results; thus, they are not currently recommended for routine clinical diagnosis [51].

NSAIDs that are potent COX inhibitors, or those that preferentially inhibit this enzyme, are the most common medications resulting in cross-reactions and numerous ADR in hypersensitive individuals. Therefore, the primary clinical recommendation for these patients is to avoid these drugs [52]. In contrast, selective COX-2 inhibitors (such as celecoxib and etoricoxib) appear to be better tolerated and may be used as a safe therapeutic option for most patients. Research has demonstrated that a substantial majority—over 90%—of patients tolerate etoricoxib very well, with an ADR rate of 7.1%. For celecoxib, the reaction rate was 11.1%, and all observed reactions were mild. These findings support the administration of these medications in such cases, provided a cautious oral provocation test is performed to exclude cross-reactivity definitively [53].

Complementary and Alternative Medicine (CAM) provides a diverse array of non-pharmacological modalities for pain modulation, which is of paramount importance for patients presenting with NSAID hypersensitivity. Physical interventions include thermal therapy (cryotherapy and thermotherapy), manual therapy (massage), physiotherapy, Transcutaneous Electrical Nerve Stimulation (TENS), acupuncture, and yoga. Furthermore, psychological interventions—specifically Cognitive Behavioral Therapy (CBT), mindfulness-based stress reduction, guided imagery, and music therapy—serve as effective adjuncts. These modalities are characterized by a favorable safety profile, minimal adverse effects, and cost-effectiveness in attenuating pain intensity [54,55].

In clinical practice, low-dose paracetamol (<500 mg) may be utilized as an alternative analgesic because of its neutral pH and weak COX-1 inhibition, which prevents the gastrointestinal toxicity, platelet dysfunction, and bronchospastic risks associated with acidic NSAIDs [56]. However, its administration is contingent upon demonstrated tolerance during provocation challenges. While often tolerated, paracetamol may elicit hypersensitivity reactions through either non-immunological COX-1 inhibition or, less frequently, via IgE-mediated selective allergy [57].

When non-opioid options are exhausted, opioids may be indicated; however, due to documented cases of hypersensitivity, an opioid provocation challenge is recommended before therapeutic use [58]. Nefopam, a non-opioid, centrally acting analgesic, represents a viable alternative. Structurally related to the antihistamine diphenhydramine and the antimuscarinic orphenadrine, nefopam does not inhibit COX-1 activity, making it safe for NSAID-hypersensitive cohorts. Its analgesic efficacy is comparable to that of NSAIDs, yet it lacks the respiratory depressive effects associated with opioids, thereby demonstrating a superior safety profile [59].

One component of managing aspirin hypersensitivity is ASA desensitization. This procedure involves rapid oral administration of escalating ASA doses, typically 0.1–10 mg. Dosing protocols are individualized for each patient, with increments administered at intervals of 10 to 30 min [60].

Desensitization is indicated for patients with NERD to alleviate sinonasal symptoms, prevent the recurrence of nasal polyps, and reduce reliance on systemic corticosteroids [61]. Furthermore, it is indicated for patients requiring chronic antiplatelet therapy following stent implantation who present with NIUA [62]. Conversely, the efficacy and appropriateness of aspirin desensitization in patients with NECD remains a subject of clinical controversy [63].

Metamizole is a potent analgesic and antipyretic agent widely utilized in clinical practice. However, its safety profile remains a subject of significant controversy due to the potential risk of developing agranulocytosis—a severe adverse effect characterized by a neutrophil count of less than 500/µL. Consequently, these safety concerns have led to stringent distribution restrictions in various countries. The underlying pathophysiology is most likely mediated by an immunological mechanism involving antibodies that bind specifically to the drug or its metabolites, ultimately leading to neutrophil destruction [64].

Notable variations in the incidence of metamizole-induced agranulocytosis (MIA) have been observed across ethnic groups; individuals of British, Irish, and Scandinavian descent appear to be more susceptible than individuals from other populations. Based on these observations, some studies suggest that these populations may share common genetic characteristics and that specific HLA allele variants may predispose them to metamizole-induced agranulocytosis [65]. At present, however, there is insufficient evidence to substantiate these potential genetic associations definitively, and further research in this area is warranted [66].

## 5. Conclusions

NSAIDs are one of the most commonly used drugs to relieve pain that are available over the counter, so hypersensitivity to them should be a significant issue in scientific research. Further research should focus on finding a new, quicker marker of NSAID hypersensitivity, because currently effective markers are highly labor-intensive, typically requiring a minimum of two days for completion, and demand stringent monitoring due to the risk of inducing severe iatrogenic bronchospasm. Additionally, focusing on ontogenic factors, such as gene polymorphisms, may be useful and create an opportunity to discover new screening methods for NSAID hypersensitivity.

NSAID hypersensitivity may express itself in diverse clinical symptoms. It recognizes 5 phenotypes, but a common feature of cross-intolerance (non-immunologic) phenotypes is the relative tolerance to selective COX-2 inhibitors, which may serve as safer therapeutic alternatives in these patients and so finding a new COX-2 selective inhibitors may extend therapeutic options, because they appear to be significantly better tolerated and may be utilized as a safe therapeutic option for the majority of patients.

Such a condition may cause patients to fear all forms of medication, so exploring their quality of life should be important to doctors. Additionally, educating patients in the doctor’s office should be an important part of the diagnostic process. As previously stated, the prevalence of NSAID hypersensitivity is higher in patients with bronchial asthma and nasal polyps. Finding different connections between NSAIDs hypersensitivity and patients’ lifestyle or environmental impact can indicate at-risk patients.

## Figures and Tables

**Table 1:** Key mediators and factors influencing NSAID hypersensitivity reactions.

Level of Mediation	Type of Reaction	References
Enzymatic Shunting	Blockade of the COX-1 enzyme leading to 5-LOX pathway overactivation; decreased synthesis of protective PGE2	[18,19]
Eicosanoid Imbalance	Overproduction of CysLTs, increased levels of LTE4 in urine during reactions.	[18,19,21]
Cellular Activation	Massive release of mediators from mast cells and eosinophils; elevated levels of mast cell tryptase.	[18,19,21]
Receptor Expression	Increased expression of high-affinity leukotriene receptors (CysLT1 and CysLT2) in the respiratory mucosa.	[18,19]
Genetic Predisposition	Polymorphisms in genes regulating leukotriene production and high-affinity IgE receptors (e.g., FcεR1β E237G and FcεR1γ 237G)	[20]

**Table 2:** Comparison of NSAID hypersensitivity phenotypes.

Phenotype	Type of Reaction	Typical Onset After NSAID Intake	Main Clinical Manifestations	Underlying Conditions	Cross-Reactivity Pattern	Tolerance to Paracetamol/COX-2 Inhibitors
NERD	Non-allergic	30 min–3 h	Bronchospasm, wheezing, dyspnea, nasal congestion, rhinorrhea, conjunctival symptoms	Asthma, chronic rhinitis, CRSwNP	cross-reactive (multiple COX-1 inhibitors)	Usually tolerated; reactions are possible at high doses
NECD	Non-allergic	1–6 h	Exacerbation of urticaria/angioedema	Chronic spontaneous urticaria	cross-reactive (multiple COX-1 inhibitors)	Usually tolerated; reactions are possible at high doses
NIUA	Non-allergic	Minutes–a few hours	Acute urticaria and/or angioedema	None	cross-reactive (multiple COX-1 inhibitors)	Usually tolerated; reactions are possible at high doses
SNIUAA	Allergic (immunologic, IgE-mediated)	Minutes–1 h (up to 6 h)	Urticaria, angioedema, anaphylaxis	None required	selective (single NSAID or related group)	Generally, well-tolerated
SNIDHR	Allergic (T-cell mediated)	Hours–days	Maculopapular exanthema, fixed drug eruptions, contact dermatitis, SCARs	None required	selective (single NSAID or related group)	Generally, well-tolerated

## Data Availability

No new data were created or analyzed in this study. Data sharing is not applicable to this article.
